# Analyzing pathogen suppressiveness in bioassays with natural soils using integrative maximum likelihood methods in R

**DOI:** 10.7717/peerj.2615

**Published:** 2016-11-03

**Authors:** Björn C. Rall, Ellen Latz

**Affiliations:** 1 German Centre for Integrative Biodiversity Research (iDiv) Halle-Jena-Leipzig, Leipzig, Germany; 2 Institute of Ecology, Friedrich-Schiller University Jena, Jena, Germany; 3 Department of Aquatic Ecology, Netherlands Institute of Ecology (NIOO-KNAW), Wageningen, The Netherlands; 4 Department of Terrestrial Ecology, Netherlands Institute of Ecology (NIOO-KNAW), Wageningen, The Netherlands; 5 Department of Animal Ecology, J.F. Blumenbach Institute of Zoology and Anthropology, Georg-August-University Göttingen, Göttingen, Germany

**Keywords:** Infected control treatments, Maximum likelihood estimation, Ordinary differential equation, Mono-molecular infection model, Biodiversity, Soil resistance, R, bbmle, DeSolve, Programming manual

## Abstract

The potential of soils to naturally suppress inherent plant pathogens is an important ecosystem function. Usually, pathogen infection assays are used for estimating the suppressive potential of soils. In natural soils, however, co-occurring pathogens might simultaneously infect plants complicating the estimation of a focal pathogen’s infection rate (initial slope of the infection-curve) as a measure of soil suppressiveness. Here, we present a method in R correcting for these unwanted effects by developing a two pathogen mono-molecular infection model. We fit the two pathogen mono-molecular infection model to data by using an integrative approach combining a numerical simulation of the model with an iterative maximum likelihood fit. We show that in presence of co-occurring pathogens using uncorrected data leads to a critical under- or overestimation of soil suppressiveness measures. In contrast, our new approach enables to precisely estimate soil suppressiveness measures such as plant infection rate and plant resistance time. Our method allows a correction of measured infection parameters that is necessary in case different pathogens are present. Moreover, our model can be (1) adapted to use other models such as the logistic or the Gompertz model; and (2) it could be extended by a facilitation parameter if infections in plants increase the susceptibility to new infections. We propose our method to be particularly useful for exploring soil suppressiveness of natural soils from different sites (e.g., in biodiversity experiments).

## Introduction

Pathogen infection assays are a standard method for estimating plant resistance to pathogens, induced systemic resistance in plants, the effect of artificial or natural plant protectants (e.g., plant beneficial bacteria), and a soil’s suppressive potential. Such bioassays are built out of a soil or substrate inoculated with a pathogen and a pathogen sensitive plant. Data is collected at just a single point in time (Maurhofer et al., 1994; Pierson & Weller, 1994; Postma et al., 2008) or at multiple points in time (e.g., Postma et al., 2008; Hanse et al., 2011; Latz et al., 2012; Latz et al., 2016). Remarkably, in the latter case often only one single point in time is chosen for evaluation (e.g., Postma et al., 2008; Hanse et al., 2011; Latz et al., 2012), or the increase from one to the next point in time is evaluated (Kushalappa & Ludwig, 1982). However, disease progression is more precisely described by classical growth curve models (Neher & Campbell, 1992). Out of the plethora of growth models (Paine et al., 2012), the mono-molecular model has often been used to describe bioassays with soil-borne pathogens (Stanghellini et al., 2004; Wilson et al., 2008). The mono-molecular infection model describes the disease progression (the change of infections over time) with an initial linear increase of infections (the infection rate), followed by a saturation (given by the maximum number of infectable plants, also known as carrying capacity or asymptotic growth).

**Figure 1:**
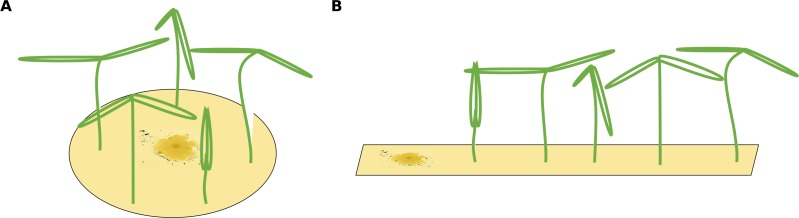
Two different possible setups for infection treatments. (A) The circular setup with a centered pathogen surrounded by plants may lead to a steep linear infection scenario as all plants are probably infected by the source pathogen at more or less the same time. (B) Only the linear spatial assembly allows for a consecutive infection of plants resulting in a linear increase that can be modeled by the mono-molecular infection model.

**Figure 2:**
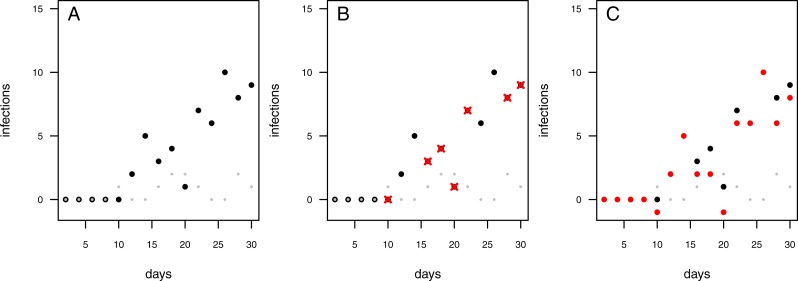
Possibilities to deal with infections observed in control treatments. Each data point represents an independent experimental pot. Grey dots: control pots (without having added the pathogen); black dots: treatment pots (containing added pathogens). (A) Infected controls are ignored and treatment data remain uncorrected. (B) In case the control pot showed an infection the respective treatment data are excluded (red crosses). (C) The treatment data are “corrected” by subtracting the number of infections in the control from the number of infections in the treatment (red dots; note that this may lead to some negative measures of infection).

The infection rate was suggested to be the most important parameter for determining pathogenicity (Raaijmakers et al., 2009). However, when estimating a soil’s suppressive potential, the time until infections occur (resistance time) might be even more important since pathogen inhibition occurs largely during pathogen growth. Actually, only a few experimental setups allow the investigation of both, infection rate and resistance time. To measure an infection rate it is necessary to use a system with multiple plant individuals (Fig. 1B) where plants can be infected one after another (i.e., measuring a time-series). In such experiments, the pathogen inoculant can be applied in different ways: (i) equally distributed application, i.e., homogeneously mixed in the soil or growth-substrate, or (ii) single point application (where pathogen spread can be assessed; Fig. 1). If a pathogen is homogeneously distributed in the plant growth substrate, it is possible to measure the number of infected plants over time. The measured infection rate, however, would not represent the infection rate per se but rather the resistance variance of the plant community to the pathogen. The same problem occurs if a pathogen is applied to one location in the substrate and plants are planted at equal distances around the inoculum (Fig. 1A). Linear spatial designs (Fig. 1B) have the potential to estimate the correct infection rate in addition to the resistance time, whereas the further mentioned approaches solely allow to estimate the resistance time. Hence, it is important to keep in mind that the design determines the hypothesis that can be tested. Another difficulty in performing pathogen infection assays occurs if natural field soils are used as substrate (e.g., Mendes et al., 2011; Latz et al., 2012; Latz et al., 2016). Here, in addition to the applied pathogen, other unknown pathogens may already exist in the soil and may increase the number of infected plants. To cope with this problem, control treatments may be used to reveal the occurrence of natural soil inhabiting pathogens. If controls show infections, (i) these infections might be ignored if they are evaluated as statistically not relevant (Fig. 2A), (ii) the treatments where the corresponding controls showed infections may be excluded from further analyses (Fig. 2B), (iii) the treatments may be linearly corrected by simply subtracting the total amount of infectable plants by the infections that occurred in the control (Fig. 2C). The third approach may lead to erroneous results in non-linear analyses as shown for functional response models (McCoy, Stier & Osenberg, 2012). However, none of these approaches are desirable as they may lead to a bias in single infection rate measures (due to ignoring or wrongly correcting infections of a naturally occurring pathogen) and the loss of data (exclusion of treatments where the corresponding control was infected).

Here, we present an alternative approach that incorporates infections caused by any additional pathogens in the system by using a two pathogen mono-molecular infection model inspired by the competition model for logistic growth (Lotka, 1925; Volterra, 1926). This two pathogen mono-molecular model is an ordinary differential equation system with two equations. Systems with two equations are hardly analytically integrable to a single equation describing the progress of infections over time, therewith preventing the use of standard linear or non-linear fitting algorithms. To overcome this limitation, we applied a numerical integration routine (Soetaert, Petzoldt & Setzer, 2010) combined with a maximum likelihood optimizer (Bolker & Team, 2016) to fit our model to data. Our method allows for the use of natural soils (i) already contaminated with naturally occurring pathogens, and (ii) from different origins and habitats, while allowing for accurate evaluation of pathogenicity and plant resistance patterns in the field.

## Methods

### Simulations

We solved the differential equation systems (Eqs. (2) & (3)) using the lsoda()-function (version 1.13; references: Soetaert & Herman, 2008; Soetaert, Petzoldt & Setzer, 2010) in R (R Core Team, 2016). The time-series length was set to 30 days with a temporal resolution of 0.01 days. *I*_max_ was fixed to 10 plants. We simulated two different scenarios; scenario 1: the natural pathogen has lower infection rates (0.001 ≤ *r*_control_ ≤ 0.1; 0.1 ≤ *r*_treatment_ ≤ 0.5) and occurs earlier in the time series as the treatment pathogen (1 ≤ *t*_0_control__ ≤ 5; 5 ≤ *t*_0_treatment__ ≤ 10); and scenario 2: the natural pathogen has comparable infection rates (0.01 ≤ *r*_control_ ≤ 0.1; 0.01 ≤ *r*_treatment_ ≤ 0.1) to the experimentally added pathogen and occurs later in the time series (5 ≤ *t*_0_control__ ≤ 10; 1 ≤ *t*_0_treatment__ ≤ 5). We draw all infection rates, *r*, and time of first infections, *t*_0_, from uniform distributions.

After simulating the time series, we randomly sampled four data-points for each full time-point (i.e., *t* = 1, 2, …, 30) assuming a binomial distribution with a size of *I*_max_ and a probability of the simulated number of infections at time *t* divided by *I*_max_ resulting in 120 independent data points for each simulated infection assay. Additionally, we simulated one or four consecutive time series resulting in 30 data points of one experimental unit (temporal autocorrelated) and 120 of four experimental units (each time series contains 30 temporal autocorrelated data points). We repeated this simulation of data 1,100 times for each scenario. We excluded model fits for both, the one-pathogen model and the two-pathogen model, if the fitting of one or the other failed and used the first 1,000 results of the cleaned data set.

### Statistical analyses

We analyzed the simulated data using an iterative maximum likelihood algorithm (function mle2() from the package **bbmle** version 1.0.18; references: Bolker, 2008; Bolker & Team, 2016) to fit Eqs. (2) & (3) to the data using R (R version 3.3.0; reference: R Core Team, 2016). See the Supplemental Information 2 for an in-depth description of the methodology.

We saved all results for the one-pathogen model fittings and the two-pathogen model fittings for each scenario and each setting (independent, one time series and four time series). Subsequently, we analyzed the *log*_10_-ratio of the fitted parameters to the initially simulated values. The starting values for infection rates where set to 50% of the simulated value and for resistance time to 75% of the simulated value. In scenario 2, the starting values for infection rates where set to 50% of the simulated value and for resistance time to 0.5 days of the simulated value.

### Empirical example

The empirical data set used as an example (Latz et al., 2016) contains control data (one consecutive time series) and treatment data (three consecutive time series). All time series have a starting value of zero for infections and measurements were taken on day 2, 4, 6, and 10. We analyzed the data as described above and in the manual. We applied additional changes to the default methodology and settings (Latz et al., 2016): (1) we decreased the step size of the numerical solver to 0.025 to ensure that eventually high infection rates (resulting in an extreme slope) are calculated precisely; (2) we allowed for 10.000 iteration steps of the mle2() function; (3) we repeated each analyses 100 times with randomized starting values, sampled out of a uniform distribution to avoid finding a local optimum (Bolker, 2008; Latz et al., 2016); (4) we selected the results with the lowest AIC out of the 100 fits.

The starting values for infection rates, *r*, ranged from 0.0001 to 3 for the control, and from 0.01 to 15 for the treatment. The starting values for resistance time, *t*_0_, ranged from 0.001 to 0.8 x day of first infection, for both, the control and the treatment.

## Results and Discussion

### The Model

The mono-molecular infection model (Raaijmakers et al., 2009; Paine et al., 2012) describes the increase of infections in a (plant) community over time, *dI* *dt*^−1^, by:

(1) \begin{eqnarray*} \frac{dI}{dt} =r \left( {I}_{\mathrm{max}}-I \right) \end{eqnarray*}

with *r* [time^−1^] being the infection rate and *I*_max_ [Infected (Plants) Area^−1^] being the maximum number of potentially infectable plants.

The infection of the first plant is not necessarily instantaneous, but depends on the resistance of the soil and the plants to the pathogen, leading to a lag phase at the beginning of the experiment. To account for this mechanism, we extend the mono-molecular infection model by the resistance time, *t*_0_:

(2) \begin{eqnarray*} \frac{dI}{dt} = \left\{ \begin{array}{@{}ll@{}} \displaystyle 0\hspace*{10.00002pt}&\displaystyle \text{if}~t\lt {t}_{0}\\ \displaystyle r \left( {I}_{\mathrm{max}}-I \right) \hspace*{10.00002pt}&\displaystyle \text{if}~t\geq {t}_{0}. \end{array} \right. \end{eqnarray*}

**Figure 3:**
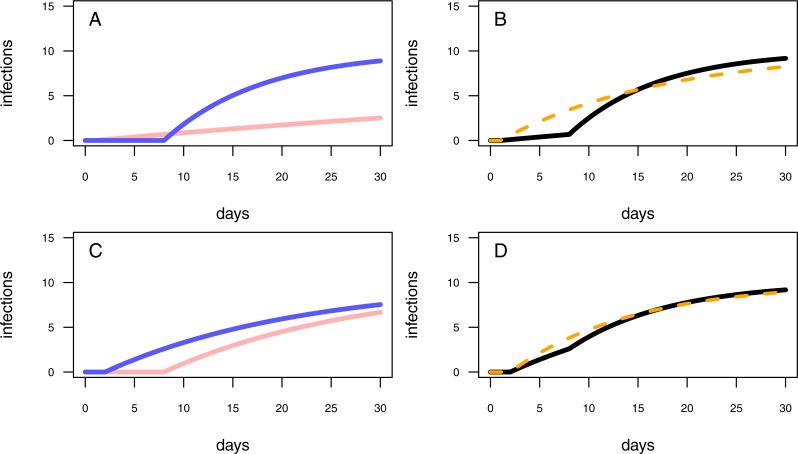
Different model configurations. (A) The one-pathogen model with two different settings of parameter values (light red line: *r* = 0.01 & *t*_0_ = 1; blue line: *r* = 0.1 & *t*_0_ = 8). (B) The two-pathogen model (black line) incorporates the parameter values of (A) and the resulting curve appears slightly above the curve of the one-pathogen model (blue line in (A)) Using a one-pathogen model to fit the black line will result in a different parameter estimation (dashed orange line, note that the dashed line is not a fitting result but a hypothetically statistical result of a wrongly chosen model). (C) The one-pathogen model with two different settings of parameter values (light red line: *r* = 0.05 & *t*_0_ = 8; blue line: *r* = 0.05 & *t*_0_ = 2). (D) The two-pathogen model (black line) incorporates the parameter values of (C) and the resulting curve appears slightly above the curve of the one-pathogen model (blue line in (C)) at a late stage of the experiment. Hypothetically using a one-pathogen model to fit may result in the orange model fit (note that the dashed line is not a fitting result but a hypothetically statistical result of a wrongly chosen model).

Below *t*_0_ new infections are zero whilst above, the occurrence of new infections follow the mono-molecular infection model. We will refer to this model as one-pathogen model (Figs. 3A, 3C).

In experiments using natural soils, natural occurring pathogens may be responsible for additional infections during the experimental trial. To correct for those infections, we extend the one-pathogen model to a two-species mono-molecular infection model, inspired by the two-species competition growth model (Lotka, 1925; Volterra, 1926):

(3) \begin{eqnarray*}\begin{array}{@{}l@{}} \displaystyle \frac{d{I}_{p}}{dt} = \left\{ \begin{array}{@{}ll@{}} \displaystyle 0\hspace*{10.00002pt}&\displaystyle \text{if}~t\lt {t}_{{0}_{p}}\\ \displaystyle {r}_{p} \left( {I}_{\mathrm{max}}-({I}_{p}+{I}_{c}) \right) \hspace*{10.00002pt}&\displaystyle \text{if}~t\geq {t}_{{0}_{p}}, \end{array} \right. \cvskip[6pt]\\ \displaystyle \frac{d{I}_{c}}{dt} = \left\{ \begin{array}{@{}ll@{}} \displaystyle 0\hspace*{10.00002pt}&\displaystyle \text{if}~t\lt {t}_{{0}_{c}}\\ \displaystyle {r}_{c} \left( {I}_{\mathrm{max}}-({I}_{p}+{I}_{c}) \right) \hspace*{10.00002pt}&\displaystyle \text{if}~t\geq {t}_{{0}_{c}}, \end{array} \right. \end{array}\end{eqnarray*}

where *I*_*p*_ is the number of infected plants due to the pathogen, *I*_*c*_ is the number of infected plants in the control; *r*_*p*_ and *r*_*c*_ are the infection rates of the pathogen and the control treatment, respectively; and *t*_0_*p*__ and *t*_0_*c*__ are the resistance times of the pathogen and the control treatment, respectively. We will refer to this model as two-pathogen model.

Below, we will give two examples of different model-parameter combinations, based on two different biological examples. We will graphically deduce how using the one-pathogen model to fit the two pathogen scenario may lead to hypothetically wrong results.

First, we assume a high infection rate *r*, and an experimental pot showing a high resistance time, *t*_0_. This will result in a first half of the experiment without any infections while in the second half of the experiment the plants will become infected rapidly (Fig. 3A, blue line). We interpret in this case an experimentally added pathogen (treatment pathogen) being inoculated in a defined distance to the seedlings. The soil is showing high suppressivess and/or highly resistant plants (high resistance time), but with the pathogen being highly abundant, it is able to infect plants rapidly after the first infection (high infection rate). However, this scenario presumes sterile soil prior to having added a treatment pathogen, whereas natural soils might be already contaminated by naturally occurring pathogens. A contaminated control pot without an experimentally added pathogen may show early infections followed by a shallow increase of infections over time (Fig. 3A, light red line). The combined progression of infections over time in a contaminated treatment pot is more complex than that of assuming only treatment pathogens being present, with showing a shallow increase of infections at low densities and a steep increase of infections in the second half of the experiment (Fig. 3B, black line). Applying the one-pathogen model to estimate the resistance time and infection rate would lead to a misleading fit (Fig. 3B, dashed orange line).

Second, we assume the plants having a rather low resistance time, *t*_0_, and the pathogen being less aggressive (low infection rate, *r*; Fig. 3C, blue line. Here, we assume a perfectly sterile experiment for both, the treatment and the control. In this example, the control treatments should not show any infections over time. However, pathogens could also disperse into the experimental pots during the experimental trial, leading to late infections of the control (Fig. 3C, red line). This might be the case when experimental pots can not be isolated from the environment, e.g., partially open mesocosms, resulting in more than the treatment pathogen being responsible for infections (Fig. 3D, black line vs. Fig. 3C, blue line). Applying the one-pathogen model to estimate the infection parameters may lead to the correct estimation of the resistance time but to an underestimation of infection rate of the treatment pathogen (Fig. 3D, dashed orange line).

In both scenarios, the use of the one-pathogen model would lead to misleading parameter estimations. To overcome this issue the two-pathogen model should be fitted to the data.

### Statistical model evaluation

#### Independent data

We tested our model framework by simulating two separate scenarios (subsequently called scenario 1 and scenario 2). In scenario 1, naturally occurring pathogens infect seedlings earlier than the treatment pathogen, but the naturally occurring pathogens are less infectious (i.e., a lower infection rate, *r*; Figs. 3A, 3B). In scenario 2, the naturally occurring pathogens infect the seedlings later than the treatment pathogen but are similar infectious (Figs. 3C, 3D). We simulated 1000 data sets where each simulated data point represents an independent measure (i.e., the end point of a single time series) for each scenario and fitted (i) the one-pathogen model to each data set (Eq. (2)) and (ii) the two-pathogen model (Eq. (3)) to each data set. We compared the fitted parameter values (i.e., the infection rate, *r*, and the resistance time, *t*_0_) by taking the log-ratio. See methods for a detailed description of the procedure.

**Figure 4:**
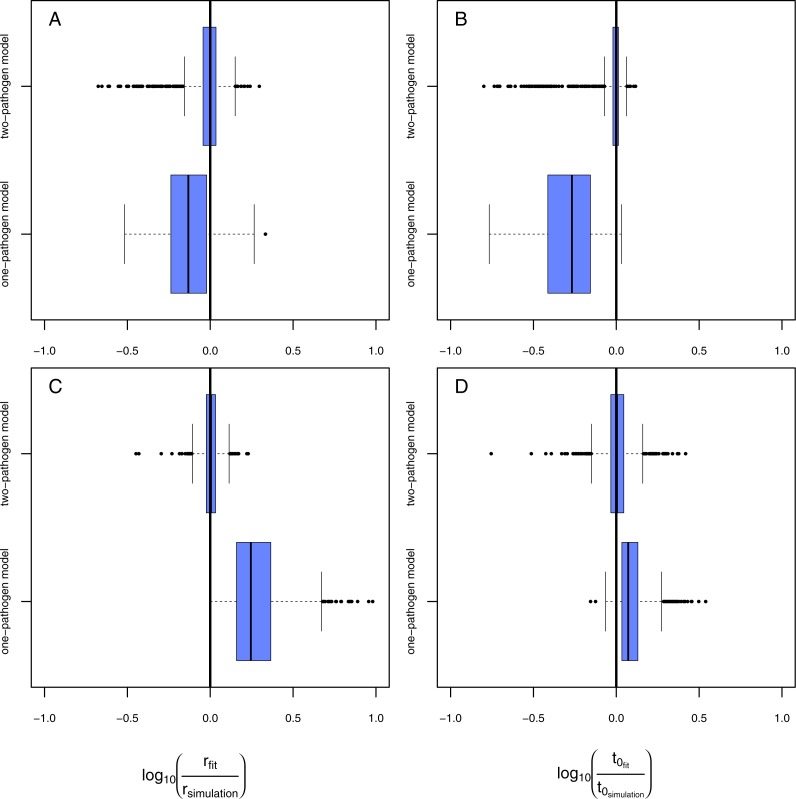
Results of the model evaluation of the one pathogen model versus the two pathogen model. The results of scenario 1 (A & B) and scenario 2 (C & D) for infections rate, *r*_*p*_, (A & C) and resistance time, *t*_0_*p*__ (B & D). The *log*_10_-ratio of the parameter fit to the real parameter used for simulating is given on the *x*-axis. If zero, the fit is perfectly reflecting the simulation, if larger than zero, the fit overestimates the real value, if smaller, the fit underestimates the real value.

**Figure 5:**
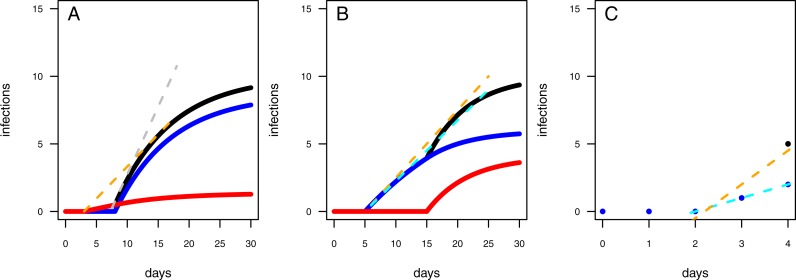
Mechanics of fitted results. (A) The two-pathogen model shows a steep increase (dashed gray line) in infections (black line) at the point in time the first infections occur due to the treatment pathogen (blue line). The naturally occurring pathogen, however, lead to infections earlier (red line) resulting in a decreased increase in infections in case using a one-pathogen model for fitting data where controls showed infections (dashed orange line). (B) The infection rates are overestimated by using a one-pathogen model (dashed orange line) to a treatment with two pathogens (black line). The real treatment infections must be lower (dashed cyan line) as only a part of the infections are caused by the treatment pathogen (blue line), the rest is caused by the control pathogen (red line). (C) The resistance time is mainly inferred by the fit using knowledge on the correct infection rates (dashed cyan line), if the infection rate is overestimated due to additional late occurring control infections (black dot) the resistance time is also overestimated (dashed orange line).

Using the one-pathogen model leads to systematic underestimation of infection rates, *r*, (Fig. 4A) whereas the two-pathogen model performs well (Fig. 4A). Also, resistance times, *t*_0_, are underestimated by the one-pathogen model (Fig. 4B) whereas the two-pathogen model predicts resistance time very precisely (Fig. 4B).

The underestimation of resistance times and infection rates nicely reflect our assumptions when fitting the one-pathogen model to the treatment (Fig. 3C). The real increase in infection is rather strong and coupled to a late first occurrence of infections (Fig. 5A, gray dashed line). But the one-pathogen model estimates a mixed increase of both, infections caused by the control and the treatment pathogens. This means that resistance time is driven by the control pathogen leading to an underestimation of infection rates (Fig. 5A, orange dashed line). The two-pathogen model, however, resolves the strong non-linear interaction between the model parameters and leads to a corrected infection curve only describing the infections by the treatment pathogen, with the correct infection rate and resistance time (Fig. 5A, blue line) that lies slightly beneath the total infection (Fig. 5A, black line).

In the second scenario (higher resistance time for the control pathogen with similar infection rates for both) the one-pathogen model overestimates the infection rates systematically (Fig. 4C). Surprisingly, resistance times are also overestimated (Fig. 4D) contrasting our expectations. In contrast, the two-pathogen model predicts simulated parameter values precisely and outperforms the one-pathogen model dramatically (Figs. 4C and 4D). Overestimation of infection rates by the one-pathogen model can be explained by additional infections later in the experiment (Fig. 5B, black line) caused by the control pathogen (Fig. 5B, red line) additionally to the infections of the treatment pathogen (Fig. 5B, blue line). These infections lead to an increase in estimated infection rates (Fig. 5B, orange line) compared to the prediction of the isolated infections of the treatment pathogen (Fig. 5B, cyan line). Interestingly, resistance times are also overestimated. This is a rather small effect and may be caused by the fact that, if the correct resistance time lies between two time steps (e.g., *t*_0_ = 2.1), the next full time step (e.g., *t*_0_ = 3) may show the first infection and the third time step the second infection we expect a rather linear increase from zero to two from time step 2 to 4 (Fig. 5C, cyan line). If control pathogens also cause infections at the third time step, the fitting algorithm will estimate a steeper increase to the cost of a higher estimated resistance time (that must still be below 3 in this example, Fig. 5C, orange line).

**Figure 6:**
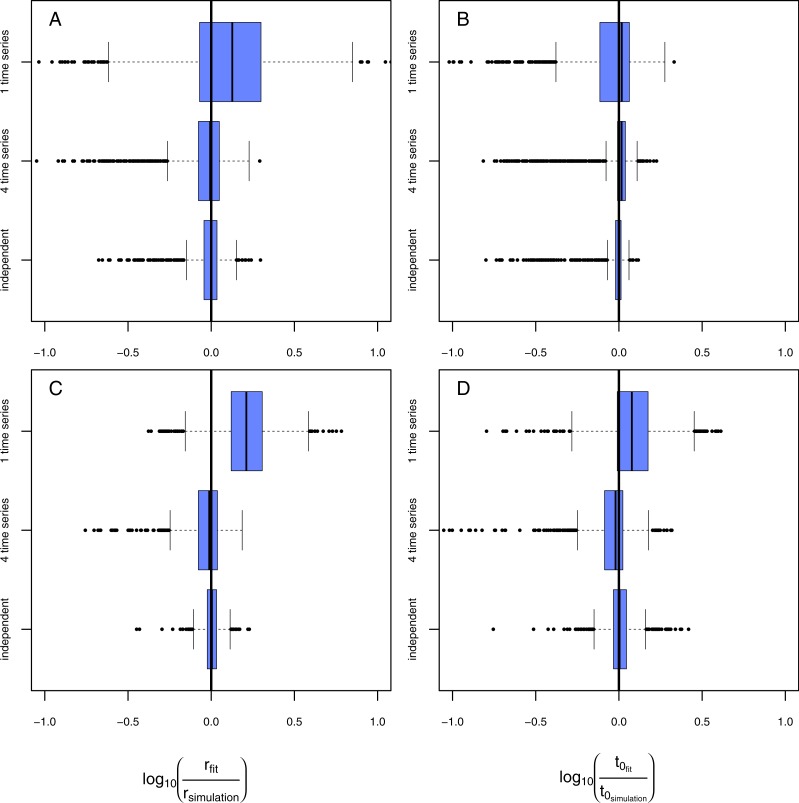
Results of the model evaluation comparing independent measures and consecutive time-series data. The results of scenario 1 (A & B) and scenario 2 (C & D) for infections rate, *r*_*p*_, (A & C) and resistance time, *t*_0_*p*__ (B & D). The *log*_10_-ratio of the parameter fit to the real parameter used for simulating is given on the *x*-axis. If zero, the fit is perfectly reflecting the simulation, if larger than zero, the fit overestimates the real value, if smaller, the fit underestimates the real value.

#### Consecutive time-series data

For the above described model comparison we used data that consisted of independent measures. This means each data point was derived from a single experimental pot that has been destructively sampled. If applying this approach to an experiment running 30 days with a resolution of one measurement per day and 4 replicates the total amount of pots that must be maintained is 120 (as in our above described analyses). Applying an additional gradient (e.g., biodiversity) would lead to a not feasible amount of experimental units. To avoid such a laborious approach, most studies measure consecutive time series where data for each temporal replicate originates from the same experimental unit. To test if our model approach is also able to fit such data adequately we simulated: (1) data of a single time series resulting in 30 measures from one experimental pot; (2) data of four time series resulting in 120 measures from four experimental pots. We only applied the two-pathogen model to the simulated data. Subsequently, we compared the deviations of model fits to the original simulated parameter values and cross-compared the quality of fits using independent data (120 measures from 120 experimental pots).

Fitting the model to data from a single time series in scenario 1 (Figs. 6A, 6B, rows: “1 time series,” naturally occurring pathogens infect the plants earlier but less strongly) leads to a slight overestimation of infection rates but in average correctly estimated resistance times. Using data from four consecutive time series (Figs. 6A, 6B, rows: “4 time series”) results in a very precise fit that is not distinguishable from the fit using independent data (Figs. 6A, 6B, rows: “independent”). In scenario 2 (Figs. 6C, 6D, rows: “1 time series,” naturally occurring pathogens infect the plants later but equally strongly) both, infection rate and resistance time, are systematically overestimated if using only one consecutive time series. Using data from four time series to estimate the parameter values statistically increases the preciseness of the fit dramatically and the results do not differ significantly from expected simulated values (Figs. 6C, 6D, rows: “4 time series”) and are only marginally worse than the results from the fit using independent data (Figs. 6C, 6D, rows: “independent”). The systematic overestimation of infection rates in both, scenario 1 and scenario 2, might be reasoned by the fact that in consecutive time series the number of infected plants can only increase opposing independent measures where infection can also decrease as they are results from independent time series (e.g., Fig. 2A).

**Figure 7:**
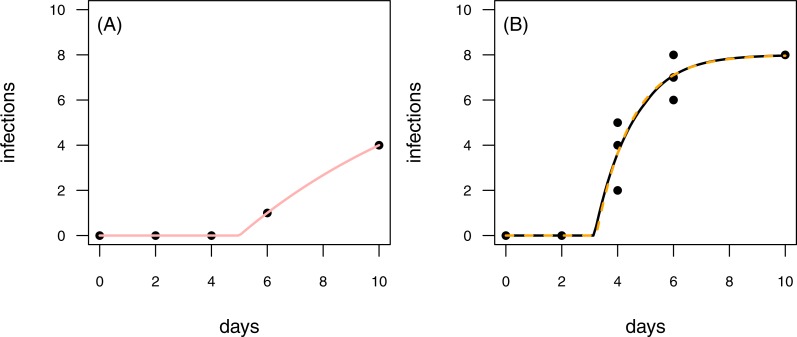
Results for the fit to empirical data. (A) The time series of the control data and the control fit, displayed as light red regression line. (B) The time series of the treatment data, having at maximum 8 plants fitted with the one-pathogen model (orange dashed regression line) as well as the two-pathogen model (black regression line).

#### Empirical examples

To provide empirical examples, we re-analyzed two data sets from  Latz et al. (2016) (see methods for details). Both data sets are consecutive time series with one control time series and three treatment time series. The first data set is characterized by infections of plants that occur later in the time series of the control than in the treatment (Fig. 7). This pattern is similar to the rules applied to scenario 2 in our model tests (see Figs. 4C & 4D and Figs. 6C & 6D). In scenario 2, the two-pathogen model predicted the real parameter values very precisely, whereas both, infection rate and resistance time were overestimated by the one-pathogen model. The results of the fit to the empirical data shows the same pattern. The infection rate of the one-pathogen model fitting is ≈0.791, whereas the infection rate measure given by the two-pathogen model is only ≈0.722. The same is true for resistance time that is ≈3.235 for the one-pathogen model and only ≈3.160 for the two-pathogen model.

**Figure 8:**
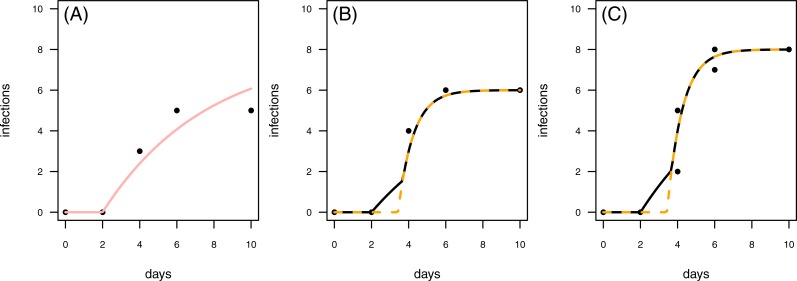
Results for the fit to empirical data. (A) The control data and the control fit, displayed as light red regression line. (B) One of the time series of the treatment data, having in total 6 plant individuals fitted with the one-pathogen model (orange dashed regression line) as well as the two-pathogen model (black regression line). (C) One of the time series of the treatment data, having at maximum 8 plants fitted with the one-pathogen model (orange dashed regression line) as well as the two-pathogen model (black regression line).

In a second empirical example we demonstrate that it is also possible to investigate time series with multiple numbers of maximum infection (*I*_max_, i.e., the maximum number of plants in the experimental pot). The treatment time series have either 6 or 8 plants in the pot that can be infected (Figs. 8B & 8C). This data set is characterized by a control where the first infection occurs on the same day as in the treatment (Fig. 8). In this case we can not clearly distinguish between scenario 1 or 2, but this example might share attributes of both. Here, infection rate as predicted by the one-pathogen model is ≈1.121 and therewith clearly higher than the infection rate that is predicted using the two-pathogen model (≈1.032). Resistance time, however, is estimated lower with the one-pathogen model (≈3.428) than with the two pathogen model (≈3.674).

### General discussion

In both scenarios, supported by the empirical examples, the two-pathogen model outclasses the one-pathogen model in predicting both, resistance time and infection rates. Moreover, our approach allows to use data from just a few consecutive time series, dramatically reducing the number of pots to be maintained. This reduced number of experimental units also allows to investigate the suppressive potential of soils in dependence of other independent variables such as biodiversity, environmental changes (e.g., a nutrient or temperature gradient), diversity and abundance of plant beneficial bacteria or pesticides (see Latz et al., 2016 as an example). To provide a relatively simple entry into our statistical method, we provide the R-code to reproduce all data and statistics presented above. Additionally, we provide an in-depth manual as additional online file (see Supplemental Information for further information). Our model approach should be easily extendable to other kinds of growth or infection models (find other growth models in reference Paine et al., 2012) to e.g., describe pathogen dispersion in larger plant communities or to include more than one treatment pathogen to estimate the competition ability of different pathogens when used together. Moreover, infected plants may be even more vulnerable to new infections. Our mathematical model could be easily adapted to such a scenario by e.g., including a facilitation term for the infection rate. However, accurately fitting such a model to empirical data requires in-depth knowledge on the identity of the pathogen infecting each plant individual, underpinning the need for more sophisticated experimental setups. Since our method is not taking any mechanisms of infection into account it is not restricted to any fungal or bacterial-pathogen bioassays but is applicable to other systems such as the dynamics of multiple herbivore pests.

The statistical method presented here is superior to classical analytic approaches such as linearization of the growth model (Neher & Campbell, 1992), estimation of infection rates by analyzing the initial increase in infections (Kushalappa & Ludwig, 1982), or arbitrary selection of a single point in time (Maurhofer et al., 1994; Pierson & Weller, 1994; Postma et al., 2008; Hanse et al., 2011; Latz et al., 2012) as it allows (1) to analyze the complete disease progression over time and (2) to correct for naturally occurring pathogens.

## Conclusions

Keystone plants as well as diverse plant communities have shown to increase pathogen suppressive potential of soils, an effect that would vanish if soils were sterilized. However, if standard approaches or the one-pathogen infection model are applied, a sterile soil is required to prevent infections by non-treatment pathogens and non-sterile soils consequently prevent the correct estimation of the pathogen suppressive potential of natural soils. This problem can be overcome by using the two-pathogen model presented in this study as it allows for the correct estimation of infection rates and resistance times. Our method will thus enable to estimate the natural suppressive potential of soils allowing an investigation of how e.g., keystone plants or specifically mixed plant communities naturally contribute to a soil resistance against pathogens.

## Supplemental Information

10.7717/peerj.2615/supp-1 Supplemental Information 1 This compressed folder contains the sub-folders “data”, “script” and “source” The folder “data” containsthe data to reproduce Figs. 4, 6, and 7: “data scenario01.csv” to “data scenario06.csv”, “real.data.1.csv” and “real.data.2.csv”. The folder “script” contains the script files “scenario01.r” to “scenario06.r” thatallow for reproducing the data shown in Figs. 4 & 6. The folder “script” contains the scriptfiles “figure07.r” & “figure08.r” allowing to reproduce the statistics shown in Figs. 7 & 8. The folder “source” contains the R-source files “infections.models.r” and “infection.nll.r” that are required to run the script files.

10.7717/peerj.2615/supp-2 Supplemental Information 2 This document includes an in-depth description on how to apply the method presented in this study in R Including how to create regression lines, trouble shooting, how to use the functions if there are differentImax, and an in-depth description of the source files.

10.7717/peerj.2615/supp-3 Supplemental Information 3 This compressed folder includes all necessary data, scripts and source files to reproduce the statistics and plots from the manual

## Additional Information and Declarations

### Data Availability

The following information was supplied regarding data availability: The raw data and R-code have been supplied as Supplemental Files.
