# Supplementary material for: Analyzing pathogen suppressiveness in bioassays with natural soils using integrative maximum likelihood methods in R
Source: PeerJ. 2016 Nov 3;4:e2615. doi: 10.7717/peerj.2615 (PMC5101589; doi:10.7717/peerj.2615)
Supplement: Supplemental Information 2 — Including how to create regression lines, trouble shooting, how to use the functions if there are differentImax, and an in-depth description of the source files. [file peerj-04-2615-s002.pdf]

Manual: Analyzing pathogen suppressiveness in bioassays with natural soils using integrative maximum likelihood methods in R

Björn C. Rall[1,2,3,4] and Ellen Latz[1,2,5]

[2]**Institute of Ecology, Friedrich Schiller University Jena**
[1]**German Centre for Integrative Biodiversity Research (iDiv) Halle-Jena-Leipzig, 04103 Leipzig, Germany**
[3]**Department of Aquatic Ecology, Netherlands Institute of Ecology (NIOO-KNAW)**
[4]**Department of Terrestrial Ecology, Netherlands Institute of Ecology (NIOO-KNAW)**
[5]**Department of Animal Ecology, J.F. Blumenbach Institute of Zoology and Anthropology, Georg-August-Universität Göttingen**

ABSTRACT

In this manual you will get a in depth description how to fit the two-pathogen model to data using R (R Core Team, 2016), with the packages **lsoda** (Soetaert and Herman, 2009; Soetaert et al., 2010) and **bbmle** (Bolker, 2008; Bolker and Team, 2016). Additionally, this manual includes a description how to add regression lines to data, a trouble shoot and an in-depth description of the underlying functions.

Keywords: infected control treatments, maximum likelihood estimation, ordinary differential equation, numerical simulation, biodiversity, soil resistance, bioassay, R, bbmle, deSolve, Manual

APPLICATION

Before starting the analyses, the packages **deSolve** (Soetaert and Herman, 2009; Soetaert et al., 2010) and **bbmle** (Bolker, 2008; Bolker and Team, 2016) must be loaded using `library()` or `require()`; the source files (infection.models.r and infection.nll.r) must be loaded using `source()`.

The Ordinary differential equations

The aim is to fit rather complex Ordinary Differential Equation systems (ODE) to data. The first equation we will use is the one pathogen model. Here the change of infections over time $dIdt^{-1}$, is described by:

$$\frac{dI}{dt} = \begin{cases} 0 & \text{if } t < t_0 \\ r\,(I_{max} - I) & \text{if } t \geq t_0. \end{cases} \tag{1}$$

with r [time^{-1}] being the infection rate and I_{max} [Infected (Plants) Area^{-1}] being the maximum number of potentially infectable plants, an t_0 is the resistance time.

The two-pathogen model can be written as:

$$\frac{dI_p}{dt} = \begin{cases} 0 & \text{if } t < t_{0_p} \\ r_p\,(I_{max} - (I_p + I_c)) & \text{if } t \geq t_{0_p}. \end{cases}$$

$$\frac{dI_c}{dt} = \begin{cases} 0 & \text{if } t < t_{0_c} \\ r_c\,(I_{max} - (I_p + I_c)) & \text{if } t \geq t_{0_c}. \end{cases} \tag{2}$$

where I_p is the number of infected plants due to the treatment pathogen, I_c are the number of infected plants in the control; r_p and r_c are the infection rates of the pathogen and the control treatment, respectively; and t_{0_p} and t_{0_c} are the resistance time of the pathogen and the control treatment, respectively.

Please see the main article for further details.

Parameter estimation

First, the control treatments (Fig. 1a) must be fitted using the monomolecular model including a lag phase (eqn (1)) using the `mle2()` function for general maximum likelihood fits from the package **bbmle** (Bolker and Team, 2016). The `mle2()` is used to minimize the negative likelihood function `mon.inf.lag.1p.nll()` from the infection.nll.r source file (see Bolker and Team, 2016, for detailed information on using the mle2() function). We need to supply the arguments `start`, containing a list of `r` and `t0` (the start values of the optimization process); `data`, a list with the headers `nI` (the number of infected plants in the experiment), `tps` (the **time**points from the experiment), and `Imax` (if the number of total plants differs between experimental units); `fixed`, a list containing the headers `steps` (the length of a time step that should be simulated by the integration routine; this is set to 0.1 as default and does not have to be supplied), `Imax`, (a single value for the maximum of potentially infectable plants in the experiment, only needed if not supplied as data), and `tracing` (if set to 1, the trace including the negative likelihood and the parameters is displayed in the console, useful for error checking, default to 0):

```
R> fit.control = mle2(mon.inf.lag.1p.nll,
+    start = list(r = 1, t0 = 1),
+    data = list(nI = y, tps = x1, Imax = x2),
+    fixed = list(steps = .1, Imax = x2, tracing = 0)
+  )
```

Note that the initial values are just placeholders and might be adapted, but see the section ”Examples” for details. `Imax` must only appear once, either in the data list or in the fixed list in dependence if `Imax` is only a fixed single value or a set of data of the same length as `y` and `x1`. Second, after estimating the infection parameters `r` and `t0` for the control, the two-pathogen monomolecular infection model can be fitted to the data with the experimentally added pathogen (equation 2). Again we use the `mle2()` function, but now we minimize the `mon.inf.lag.2p.nll()` function. The estimates of the control treatment are assigned to `rc` and `t0c` in the fixed argument list. The target infection parameters are still placed in the start argument but now called `rp` and `t0p`:

```
R> fit.treatment = mle2(mon.inf.lag.2p.nll,
+    start = list(rp = 1, t0p = 1),
+    data = list(nI = y, tps = x1, Imax = x2),
+    fixed = list(steps = 1,
+      Imax = x2,
+      rc = coef(fit.control)[[1]],
+      t0c = coef(fit.control)[[2]]
+    )
+  )
```

One can investigate the fitting results and statistics using the generic `summary()` function applied on the object containing fitting results (here: `fit.control` & `fit.treatment`).

EXAMPLES

The supplemental information contains R-code files. The files infection.frontend1.r and infection.frontend2.r are the front-end files containing the adaptable examples we will discuss below. These files load the source files, infection.models.r and infection.nll.r, containing all underlying functions. We begin with a detailed description of the front-end files which should be sufficient to apply our fitting method to data. Moreover, we kindly invite you to continue reading the descriptions of the source files later on (section ”Underlying Functions”). Please extract all required files from the zip-folder into one folder and keep the provided folder structure. If you prefer different organization of data and files you have to adapt the paths described below in the code. Before getting started, use e.g. `install.packages()` to install the required packages **deSolve** (Soetaert et al., 2010) and **bbmle** (Bolker and Team, 2016), but see introductory R Books and manuals for details (e.g. Bolker, 2008; Crawley, 2012). We first discuss the case of a constant number of plants in the experimental units (infection.frontend1.r).

Required packages and data

First, the required packages, **deSolve** (Soetaert et al., 2010) and **bbmle** (Bolker and Team, 2016), the source files and the data must be loaded:

```
R> library("deSolve")
R> library("bbmle")
R> setwd(/path/to/your/folder/) #please replace this according to your path
R> source("source/infection.models.r")
R> source("source/infection.nll.r")
R> sample.data = read.csv("data/sample.data.csv")
```

To investigate the data structure we use the generic `str()` function:

```
R> str(sample.data)
```

```
R> str(sample.data)
```

```
'data.frame': 40 obs. of  3 variables:
 $ treatment      : Factor w/ 2 levels "control","treatment": 1 1 1 1 1 ...
 $ time.days      : int  1 2 3 4 5 ...
 $ number.infected: int  0 0 1 1 3 ...
```

The data comprises three variables, (1) the factorial variable treatment determining if the data belongs to the control or the experimental treatment. The variable `time.days` contains the time of the measurement in days (you can switch the temporal resolution, the unit here determines the unit of the infection rate), and the variable `number.infected` containing the information of how many plants are infected (note that this must be an integer as we apply a binomial distribution later on; but see Crawley (2012) and Bolker (2008) for a detailed description on this topic). The data represents single experimental units (independent replicates, each data point represents the last measurement of a time series). Before continuing, we separate the data into two sub data sets containing just the control or just the treatment data using the subset function.

```
R> data.control = subset(sample.data, treatment == "control")
R> data.treat = subset(sample.data, treatment == "treatment")
```

Subsequently, we investigate the data graphically. For a better overview we set the graphical device to display two plotting regions using the `par()` function (the argument `mfrow` is set to `c(1,2)`, generating 2 horizontal adjacent plotting regions). Within the `plot()` functions we fix the y-axes ranges to create comparable plots (`ylim = c(0,10)`) and display the number of infections (y-axis) depending on the experimental time (x-axis):

```
R> par(mfrow=c(1,2))
R> plot(data.control$time.days,
+    data.control$number.infected,
+    ylim=c(0,10),
+    xlab="days",
+    ylab="infections control")
R> plot(data.treat$time.days,
+    data.treat$number.infected,
+    ylim=c(0,10),
+    xlab="days",
+    ylab="infections treatment")
```

The control without an experimentally added pathogen shows an early increase (day 3) of infected plants over time (Fig. 1a), but not all plants are infected during the experimental trial. The treatment with the experimentally added pathogen shows the first infections at day 4, but the increase in new infections is much steeper than in the control, and all plants might be infected (Fig. 1b).

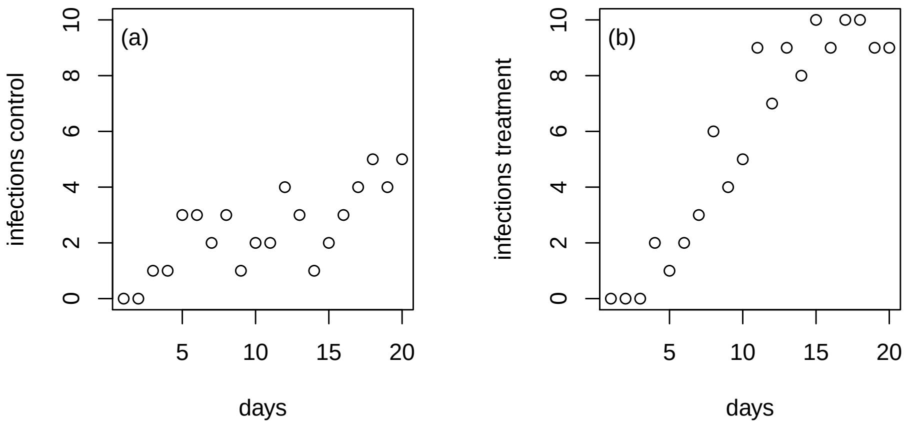

Figure 1. Two different possible setups for infection treatments. Simulated data of an infection bioassay. The data is simulated based on equations (1) and (2) using the functions `mon.inf.lag.1p()` and `mon.inf.lag.2p()`. After simulating the numeric average we applied a random number generator (`rbinom()`) using `Imax` as size of the binomial distribution and the floating average of the simulation divided by `Imax` as probability. The control data (a) was simulated using `r` = 0.025, `Imax` = 10 and `t0` = 1 and the experimental treatment (b) was simulated using `rp` = 0.19, `rc` = 0.025, `t0p` = 5.5, `t0c` = 1 and `Imax` = 10.

Analysis of the data - standard approach

First, we analyze the experimental data using the standard mono-molecular growth model (Raaijmakers et al., 2009; Paine et al., 2012). This model ignores the fact that not only the experimentally added pathogen but, in addition, other soil inherent pathogens may infect plant individuals and will be named ”wrong method” (`fit.treatment.wrong()`, note that this method is valid if the medium chosen for the bioassay is sterile or does not contain any alternative pathogens). To fit the model to the data we use the `mle2()` function from the **bbmle** package by Ben Bolker Bolker and Team (2016); Bolker (2008). The `mle2()` function requires (1) a negative log-likelihood function, here the function `mon.inf.lag.1p.nll()` discussed later (see section - ”Underlying functions”); (2) a list containing the model parameters that should be estimated (here the infection rate, `r`, and the resistance time, `t0`); (3) a list containing the data the model should be fitted to, here the first element of the list must be named `nI` (the number of infected plants) and the second element must be named `tps` (**time** **p**oint**s**, the time of the experimental data); (4) we also submit a fixed value to `mle2()`, again in a list object, containing the maximum number of potentially infected plants (all plants in the experimental unit). Note that `Imax` does not have to be a fixed value but can also be assigned in the data list if the different replicates contain different numbers of plants (see section ”Analysing data with multiple `Imax`”). Here, we start the fitting optimization with the initial guessed values of `r = 1` and `t0 = 1` (note that these values are just place-holders and might be adapted by the user,see section ”What to do if” below, moreover these values will be changed during the fitting algorithm by the optimizer `mle2()`; note also that the initial `t0` must be equal or smaller than the experimental resistance time, which according to figure 1 is at day 4); we set `Imax = 10` (the maximum number of plants in the experiment).

```
R> fit.treatment.wrong = mle2(minuslogl = mon.inf.lag.1p.nll,
+    start = list(r = 1, t0 = 1),
+    data = list(nI = data.treat$number.infected,
+      tps = data.treat$time.days),
+    fixed = list(Imax = 10)
+  )
```

After the fitting procedure, we investigate the data using the generic `summary()` function:

```
R> summary(fit.treatment.wrong)
```

```
Maximum likelihood estimation
Call:
mle2(minuslogl = mon.inf.lag.1p.nll, start = list(r = 1, t0 = 1),
    fixed = list(Imax = 10), data = list(nI = data.treat$number.infected,
        tps = data.treat$time.days))
Coefficients:
   Estimate Std. Error z value     Pr(z)
r  0.170595   0.021636  7.8849 3.148e-15 ***
t0 3.461684   0.392051  8.8297 < 2.2e-16 ***
---
Signif. codes:  0 '***' 0.001 '**' 0.01 '*' 0.05 '.' 0.1 ' ' 1
-2 log L: 49.99375
```

The output provides information on the estimates of the model, as well as statistical indicators such as standard errors and p-values (but see Bolker 2008 for a detailed discussion of the underlying statistics). The infection rate, `r`, is estimated to be 0.171 +/- 0.022 infections per day, and the resistance time, `t0`, is estimated to be 3.46 +/- 0.39 days. These values underestimate both model parameters compared to the simulation parameters ($t0_{simulation}$ = 5.5 days, $r_{simulation}$ = 0.19 infections per day).

Analysis of the data - corrected approach

Next, we apply the two-pathogen approach to the data, taking infections of the control treatments into account. In the first step we analyze the control data similarly to the treatment data shown above only with exchanging the data supply from the experimental to the control data and decreasing the starting values for the model parameters (we already saw that the first infections occurred earlier and the slope is less step than for the treatment data).

```
R> fit.control = mle2(mon.inf.lag.1p.nll,
+    start = list(r = 0.5, t0 = 0.5),
+    data = list(nI = data.control$number.infected,
+      tps =  data.control$time.days),
+    fixed = list(Imax = 10)
R> )
R> summary(fit.control)
```

```
Maximum likelihood estimation
Call:
mle2(minuslogl = mon.inf.lag.1p.nll, start = list(r = 0.5, t0 = 0.5),
    fixed = list(Imax = 10), data = list(nI = data.control$number.infected,
        tps = data.control$time.days))
Coefficients:
    Estimate Std. Error z value     Pr(z)
r  0.0292603  0.0075671  3.8668 0.0001103 ***
t0 0.4666304  2.0573508  0.2268 0.8205705
---
Signif. codes:  0 '***' 0.001 '**' 0.01 '*' 0.05 '.' 0.1 ' ' 1
-2 log L: 56.46962
```

The fitting algorithm revealed that the infection rate, `r`, is 0.029 +/-0.0076, and the resistance time, `t0`, is 0.47 +/-2.057. Both values are not significantly different from the simulated value. We will use the infection rate and the resistance time of the control to parameterize the two-pathogen model

using the mon.inf.lag.2p.nll() function (see section ”Underlying Functions”, for details). Using mon.inf.lag.2p.nll() requires some reformulation of the code, the infection rate, r, and the resistance time, t0, for the treatment ”pathogen” are now called rp and t0p and both appear in this spelling in the list of starting parameters. In addition to Imax, the list of fixed parameters contains here the infection rate of the control pathogen, rc, and the resistance time of the control, t0c. We call this values directly using the coef() function.

```
R> fit.treatment = mle2(mon.inf.lag.2p.nll,
+     start = list(rp = 0.5, t0p = 5),
+     data = list(nI = data.treat$number.infected,
+       tps = data.treat$time.days),
+     fixed = list(Imax = 10,
+       rc = coef(fit.control)[[1]],
+       t0c = coef(fit.control)[[2]])
+   )
R> summary(fit.treatment)
```

```
Maximum likelihood estimation
Call:
mle2(minuslogl = mon.inf.lag.2p.nll,
start = list(rp = 0.5, t0p = 5),
fixed = list(Imax = 10, rc = coef(fit.control)[[1]],
    t0c = coef(fit.control)[[2]]),
data = list(nI = data.treat$number.infected,
    tps = data.treat$time.days))
Coefficients:
    Estimate Std. Error z value     Pr(z)
rp  0.193377   0.035434  5.4574 4.831e-08 ***
t0p 5.831930   0.614306  9.4935 < 2.2e-16 ***
---
Signif. codes:  0 '***' 0.001 '**' 0.01 '*' 0.05 '.' 0.1 ' ' 1
-2 log L: 48.21475
```

The values estimated by the two pathogen mono-molecular model match the simulated values better than the results from the uncorrected fit. The estimated infection rate is measured to be 0.193 +/- 0.035 (rp, simulation = 0.19) and the first infection time is estimated to be 5.83 +/- 0.61 (t0p, simulation = 5.5).

Adding lines to the plot

The graphical representation of data and model fits is common practice. Simple linear regressions can be added as line to an existing plot using e.g. the generic abline() function. Our example is slightly more complex, but not much. First we have to create a vector containing values for the x-axis (time in days in our case) which will later be used to display a line in the plot. Note that non-linear lines need many values to create a smooth appearance of the line. Here we choose to create 100 x-values ranging from t=0 to t=20, the end of our experiment using the seq() function with the third argument set to length = 100 (which creates 100 evenly distributed values ranging from the minimum to the maximum value). To simulate the corresponding y-values we use the lsoda() function from the package **deSolve** (Soetaert et al., 2010). The lsoda() function builds a complex object including background information on the simulation run not needed for our purpose. To get rid of this background information, we save the object created by lsoda() as a data frame by applying the data.frame() function on the lsoda() function. lsoda() requires the starting density of the infected plants as first argument, y, here c(I = 0); the second argument is the sequence of time points the number of infected plants should be calculated for; the third argument requires the ordinary differential equation model that should be applied (mon.inf.lag.1p(), see below for detailed discussion); and fourth, the parameters of the model must be supplied, here the results of our model fit.

```
R> xvalues = seq(0,20,length=100)
R>
R> control.est =  data.frame(lsoda(y = c(I = 0),
+    times = xvalues,
+    func = mon.inf.lag,
+    parms = c(r = coef(fit.control)[[1]],
+      t0 = coef(fit.control)[[2]],
+      Imax = 10)
+  ))
```

To simulate the regression line for the correct treatment fit, we must extend the code described above to incorporate both the control as well as the treatment pathogen parameters. We again use the `lsoda()` function, but now call the `mon.inf.lag.2p()` function that allows to model two pathogens. Also, we have to provide two starting densities for the infected plants (zero infected plants by the treatment pathogen, `Ip = 0`, and zero infected plants by the natural occurring (control) pathogens, `Ic = 0`). Moreover, the parameter list must now contain five parameters, both infection rates (`rp` and `rc`), both resistance times (`t0p` and `t0c`) and again the maximum reachable number of infected plants, `Imax`.

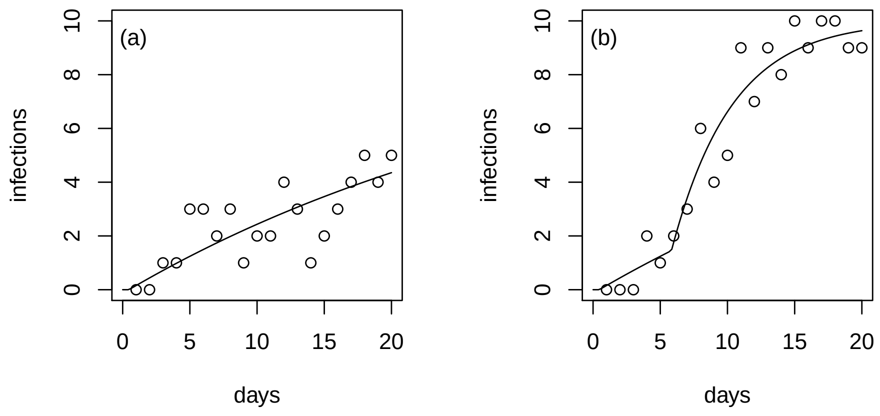

Figure 2. Infection bioassay as in figure 1. The control (a) shows less infections starting earlier in the time series compared to the treatment with the experimentally added pathogen (b). This leads to a low infection rate combined with a low resistance time visualized by the black line in (a). The early infections in the treatment are due to infections by the natural pathogen, leading to a slight increase of the fitted curve (b), at t $\sim$ 6, the treatment pathogen leads to a steep increase of the infection curve.

```
R> treat.est =  data.frame(lsoda(y = c(Ip = 0, Ic = 0),
+    times = xvalues,
+    func = mon.inf.lag.2p,
+    parms = c(rp = coef(fit.treatment)[[1]],
+      t0p = coef(fit.treatment)[[2]],
+      rc = coef(fit.treatment)[[3]],
+      t0c = coef(fit.treatment)[[4]],
+      Imax = 10)
+  ))
```

To add the lines to the plot, we again call `plot()` from above and subsequently `lines()`. Note that we have to sum both densities of infected plants (due to the treatment pathogen and the natural occurring (control) pathogen) to plot correct line (Fig. 2) for the treatment data.

```
R> par(mfrow=c(1,2))
R> plot(data.control$time.days,
+    data.control$number.infected,
+    xlim=c(0,20),
+    ylim=c(0,10),
+    xlab="days",
+    ylab="infections")
R> mtext(side=3,"(a)",line=-1.5,adj=0.03)
R> lines(control.est$time, control.est$I)
R>
R> plot(data.treat$time.days,
+    data.treat$number.infected,
+    xlim=c(0,20),
+    ylim=c(0,10),
+    xlab="days",
+    ylab="infections")
R> mtext(side=3,"(b)",line=-1.5,adj=0.03)
R> lines(treat.est$time, treat.est$Ip+treat.est$Ic)
```

WHAT TO DO IF...

... I get the

```
Warning message:
In dbinom(x = nI, prob = Isim/Imax, size = Imax, log = TRUE) :
  NaNs produced
```

Keep calm, this is just a warning message, not an error and the fitting algorithm still succeeded. Let us perform an example using the control treatment from the infection.frontend1.r (line 107 ff). We change the initial infection rate to 3, a rather high starting value:

```
R> fit.control.warning = mle2(mon.inf.lag.1p.nll,
+    start = list(r = 3, t0 = 0.5),
+    data = list(nI = data.control$number.infected,
+      tps =  data.control$time.days),
+    fixed = list(Imax = 10)
+ )
```

```
Warning messages:
1: In dbinom(x = nI, prob = Isim/Imax, size = Imax, log = TRUE) :
  NaNs produced
2: In dbinom(x = nI, prob = Isim/Imax, size = Imax, log = TRUE) :
  NaNs produced
```

```
R> summary(fit.control.warning)
```

```
Maximum likelihood estimation
Call:
mle2(minuslogl = mon.inf.lag.1p.nll, start = list(r = 3, t0 = 0.5),
     fixed = list(Imax = 10),
     data = list(nI = data.control$number.infected,
        tps = data.control$time.days))
Coefficients:
```

```
    Estimate Std. Error z value     Pr(z)
r  0.0292636  0.0075801  3.8606 0.0001131 ***
t0 0.4656128  2.0637430  0.2256 0.8215003
---
Signif. codes:  0 '***' 0.001 '**' 0.01 '*' 0.05 '.' 0.1 ' ' 1
-2 log L: 56.46962
```

The warning message appears twice, but the fit still succeeded. But we can have a closer look at the problem. To do so, we can have a look at the trace log of the fit.

... I want to see the trace of the integrative fitting process?

We continue the example by adding the element `tracing` to the fixed list. This allows to track the iteration steps in which the `NaNs` occurred and the values of the infection parameters that have been used in these steps:

```
R> fit.control.tracing = mle2(mon.inf.lag.1p.nll,
+    start = list(r = 3, t0 = 0.5),
+    data = list(nI = data.control$number.infected,
+      tps =  data.control$time.days),
+    fixed = list(Imax = 10, tracing = 1)
+  )
```

Running this code a list of negative likelihood values and infection parameters appears in the console, getting longer the more the fitting advances (to save space here in the text we truncated the output, you might have to scroll up a little in your console output to see the same):

```
...
[1] "negLL:  28.2629342646186 r:  0.0277248134455506 t0:  0.248536802570284"
[1] "negLL:  28.2627221727419 r:  0.0277248134455506 t0:  0.246536802570284"
[1] "negLL:  NaN r:  54.3011142347463 t0:  0.141490864217889"
[1] "negLL:  NaN r:  10.8824026977057 t0:  0.226327614899805"
[1] "negLL:  2897.85108207059 r:  2.19866039029758 t0:  0.243294965036188"
[1] "negLL:  582.147373888558 r:  0.461911928815957 t0:  0.246688435063465"
...
```

Clearly, the `NaNs` are produced when extremely high infection rates ($\texttt{r} \sim 50.3$ and $\texttt{r} \sim 10.88$) are tested by the optimization algorithm. This causes the `lsoda()` to fail as the increase in new infected plants is much larger than the step size of the numeric integration routine (we set the default to 0.1, which corresponds here to 0.1 days). Therefore (1) the warning message can be ignored as it applies only to unlikely high infection rates; a change in the starting parameters may avoid that the optimization algorithm randomly picks these high rates (the main example did not show any warnings); or the step size of the numerical integration routine can be decreased to increase the temporal resolution of the simulation.

... I want to change the step size of the numerical integration routine?

```
R> fit.control.steps = mle2(mon.inf.lag.1p.nll,
+    start = list(r = 3, t0 = 0.5),
+    data = list(nI = data.control$number.infected,
+      tps =  data.control$time.days),
+    fixed = list(Imax = 10, steps = 0.01)
+  )
R> summary(fit.control.steps)
```

```
Maximum likelihood estimation
Call:
mle2(minuslogl = mon.inf.lag.1p.nll,
start = list(r = 3, t0 = 0.5),
fixed = list(Imax = 10, steps = 0.01),
data = list(nI = data.control$number.infected,
tps = data.control$time.days))
Coefficients:
    Estimate Std. Error z value     Pr(z)
r  0.0292641  0.0076073  3.8468 0.0001197 ***
t0 0.4656282  2.0725455  0.2247 0.8222400
---
Signif. codes:  0 '***' 0.001 '**' 0.01 '*' 0.05 '.' 0.1 ' ' 1
-2 log L: 56.46962
```

By decreasing the temporal resolution of the underlying integration of the infection model, we got rid of the warning messages, but at the cost of speed (it will take much longer to run the model function with this settings).

ANALYSING DATA WITH MULTIPLE IMAX

In bioassays using plant seeds instead of plants not all seeds may germinate. This leads to different Imax in the different replicates. You find the example in `infection.frontend2.r`. As for the above mentioned example, we first have to set the working directory, load the required packages, source files and data:

```
R> library("deSolve")
R> library("bbmle")
R> source("source/infection.models.r")
R> source("source/infection.nll.r")
R> sample.data2 = read.csv("data/sample.data2.csv")
R> str(sample.data2)
```

```
'data.frame': 60 obs. of  4 variables:
 $ treatment      : Factor w/ 2 levels "control","treatment": 1 1 1 1 1 ...
 $ time.days      : int  1 2 3 4 5 ...
 $ number.infected: int  0 0 0 1 2 ...
 $ total.number   : int  10 10 10 12 9 ...
```

You might notice that the data set now includes the additional variable `total.number` containing the information which experimental unit has which `Imax`. We continue with separating the data sets as above:

```
R> data2.control = subset(sample.data2, treatment == "control")
R> data2.treat = subset(sample.data2, treatment == "treatment")
```

As described above, we begin by fitting the control treatment. Additionally we set the step length of the numerical integrator to 0.01 to avoid `NaNs`:

```
R> fit.control = mle2(mon.inf.lag.1p.nll,
+    start = list(r = 0.02, t0 = 0.5),
+    data = list(nI = data2.control$number.infected,
+      tps =  data2.control$time.days,
+      Imax = data2.control$total.number),
+    fixed = list(steps = 0.01)
+  )
R> summary(fit.control)
```

```
Maximum likelihood estimation
Call:
mle2(minuslogl = mon.inf.lag.1p.nll,
start = list(r = 0.02, t0 = 0.5),
fixed = list(steps = 0.01),
data = list(nI = data2.control$number.infected,
tps = data2.control$time.days,
Imax = data2.control$total.number))
Coefficients:
    Estimate Std. Error z value   Pr(z)
r  0.0060132  0.0024244  2.4803 0.01313 *
t0 2.8417380  6.3979722  0.4442 0.65693
---
Signif. codes:  0 '***' 0.001 '**' 0.01 '*' 0.05 '.' 0.1 ' ' 1
-2 log L: 47.61573
```

The most important change is that we supply the information for `Imax` to the `data` list instead of the `fixed` list. In total, the fit takes longer as in the example above. This is due to the fact that a numerical integration of a time series for each Imax must be calculated which is rather time consuming. We provide more details on this below. After fitting the controls we follow the procedure explained above and fit the treatments:

```
R> fit.treatment = mle2(mon.inf.lag.2p.nll,
+    start = list(rp = 1, t0p = 1),
+    data = list(nI = data2.treat$number.infected,
+      tps = data2.treat$time.days,
+      Imax = data2.treat$total.number),
+    fixed = list(steps = 0.01,
+      rc = coef(fit.control)[[1]],
+      t0c = coef(fit.control)[[2]])
+  )
R> summary(fit.treatment)
```

```
Maximum likelihood estimation
Call:
mle2(minuslogl = mon.inf.lag.2p.nll,
start = list(rp = 1, t0p = 1),
fixed = list(steps = 0.01,
rc = coef(fit.control)[[1]],
t0c = coef(fit.control)[[2]]),
data = list(nI = data2.treat$number.infected,
tps = data2.treat$time.days,
Imax = data2.treat$total.number))
Coefficients:
    Estimate Std. Error z value    Pr(z)
rp   0.60307    0.26499  2.2758  0.02286 *
t0p  8.27764    1.15717  7.1533 8.47e-13 ***
---
Signif. codes:  0 '***' 0.001 '**' 0.01 '*' 0.05 '.' 0.1 ' ' 1
-2 log L: 9.043275
```

UNDERLYING FUNCTIONS

Negative Likelihood Functions (infection.nll.r)

The fitting algorithm used above, `mle2()`, requires a negative log-likelihood (nll) function that will be minimized (see Bolker, 2008, for a detailed introduction). The `nll()`-function for the monomolecular infection model can be written as:

```
R> mon.inf.lag.1p.nll = function(nI,tps,r,t0,Imax,steps=0.1,tracing=0){
+    if(r <= 0 || t0 <= 0 || t0 >= min(tps[nI>0])){ return(Inf) }
+    Isim = sim.inf.1p(tps, r, Imax, t0, steps)
+    negLL = -sum(dbinom(x = nI,
+      prob =  Isim / Imax,
+      size = Imax,
+      log = TRUE))
+    if(tracing == 1) print(paste("negLL: ",negLL,"r: ",r,"t0: ",t0))
+    return(negLL)
+ }
```

The function includes seven arguments: the experimentally measured number of infected plants, `nI`; the corresponding time estimates from the experiment, `tps` (**t**ime**p**oint**s**); the infection rate, `r`; the resistance time, `t0`; the maximum number of infections possible, `Imax`, (i.e. the maximum number of host plants in the experimental unit); the desired step length of the numerical integration, `steps`, by default set to 0.1; and the tracing argument which is deactivated by default. First the function checks if either `r` or `t0` are below zero or `t0` is greater than or equal to the minimum time of an infection in the real data. If any of these queries is true, the function returns infinity. Next, the model predictions for each experimentally measured value are simulated using `sim.inf.1p()` (see section "ODE functions"). Finally, the function calculates the negative likelihood using the binomial density function `dbinom()` and returns it. We choose the binomial distribution as our data is binomially distributed (integer values for infections have a clear defined minimum and maximum number of infections, but see Bolker (2008) for a detailed introduction on this topic). The two-pathogen variant, `mon.inf.lag.2p.nll()`, is similar to the above described function and we will only discuss the differences. As this function is created to estimate the negative log-likelihood of a two pathogen system, the model parameters consist of `rp`, `rc`, `t0p` and `t0c`. Moreover, the `function sim.inf.2()` is used to calculate the number of infections from the model simulation. Also a more complex if-statement is added to ensure that at least one of `t0p` or `t0c` falls below the time the first infection occurred in the experiment (otherwise `NaNs` may be produced):

```
R> mon.inf.lag.2p.nll = function(nI, tps, rp, t0p, rc,
+    t0c, Imax, steps = 0.1, tracing = 0){
+    if(rp <= 0 || t0p <= 0 || rc <= 0 || t0c <= 0){ return(Inf) }
+    if(t0p >= min(tps[nI>0]) && t0c >= min(tps[nI>0])){ return(Inf) }
+    Isim = sim.inf.2p(tps, rp, rc, Imax, t0p, t0c, steps)
+    negLL = -sum(dbinom(x = nI,
+      prob = Isim / Imax,
+      size = Imax,
+      log = TRUE))
+    if(tracing == 1) print(paste("negLL: ",negLL, "r: ",r, "t0: ",t0))
+    return(negLL)
+ }
```

The function `sim.inf.1()` first creates an empty numeric vector (`Iout`) to store the calculated values for infections. If `Imax` is only a single value we apply the `lsoda()` function (Soetaert et al., 2010) to simulate a single time series of plant infections according to the assigned parameter values (`parms = c(r = r, Imax = Imax, t0 = t0)`) and a starting density of infected plants of unity (`y = c(I = 0)`). The numerical simulation of the integration process needs a vector of consecutive points in time. This vector consists of a sequence of consecutive values from zero to the maximum time value (`seq(0, max(tps), steps)`), and the experimental time values, `tps`. As no duplicate values should appear in the vector and the time vector should increase consecutively, we first apply the `unique()` function on the vector to delete duplicates and second, sort the vector by the `sort()` function. The ordinary differential equation system that should be integrated is given by the function `mon.inf.lag.1p()`. Please read into Soetaert and Herman (2009) to get a general introduction into the topic "solving ordinary differential equation systems in R using deSolve". After the integration the number of estimated infections are saved according to their appearance in the experimental time series using a for loop. If the experimentally data consists of more than one single value for `Imax`, the else

part of the `if`/`else`-statement is activated. First, we create three empty numeric vectors to store the the the number of infected plants, the time, and the maximum number of infectable plants in (`mres.I`, `mres.time`, `mres.Imax`). Second, we use a `for`-loop to calculate the number of infected plants for each `Imax`. Third, we save the results to `Iout` as described above using a `for`-loop, with additionally separating for each `Imax`.

```
R> sim.inf.1p = function(tps, r, Imax, t0, steps) {
+    Iout = numeric()
+    if(length(Imax) == 1){
+      mres = data.frame(lsoda(y = c(I = 0),
+      times = sort(unique(c(seq(0, max(tps), steps), tps))),
+      func = mon.inf.lag.1p,
+      parms = c(r = r,
+        Imax = Imax,
+        t0 = t0)))
+      for(tps.i in 1:length(tps)){
+        Iout[tps.i] = mres$I[mres$time == tps[tps.i]]
+      }
+    } else {
+      mres.I = numeric()
+      mres.time = numeric()
+      mres.Imax = numeric()
+      for(Imax.i in 1:length(unique(Imax))){
+        mres = data.frame(lsoda(y = c(I = 0),
+          times = sort(unique(c(seq(0, max(tps), steps), tps))),
+          func = mon.inf.lag.1p,
+          parms = c(r = r, Imax = unique(Imax)[Imax.i], t0 = t0)))
+        mres.I = c(mres.I,mres$I)
+        mres.time = c(mres.time,mres$time)
+        mres.Imax = c(mres.Imax,
+          rep(unique(Imax)[Imax.i], length(mres$time)))
+      }
+      for(tps.i in 1:length(tps)){
+    Iout[tps.i]=mres.I[mres.time == tps[tps.i] & mres.Imax == Imax[tps.i]]
+      }
+    }
+    return(Iout)
+  }
```

The function `sim.inf.2()` is similar to the function `sim.inf.1()` but models the two-pathogen system. The differences are: model parameters consist are `rp`, `rc`, `t0p` and `t0c`; `lsoda()` needs two starting values for infections at time = 0 (`c(Ip = 0, Ic = 0)`); the ordinary differential equation system is given by the function `mon.inf.lag.2p()`. The results for the total infected plants, estimated by the model, are now calculated by (`mres`$Ic + mres$`Ip`).

```
R> sim.inf.2p = function(tps, rp, rc, Imax, t0p, t0c, steps) {
+    Iout = numeric()
+    if(length(Imax) == 1){
+      mres = data.frame(lsoda(y = c(Ip = 0, Ic = 0),
+        times = sort(unique(c(seq(0, max(tps), steps), tps))),
+        func = mon.inf.lag.2p,
+        parms = c(rp = rp,
+          rc = rc,
+          Imax = Imax,
+          t0p = t0p,
+          t0c = t0c)))
+        for(tps.i in 1:length(tps)){
+          Iout[tps.i] = (mres$Ip+mres$Ic)[mres$time == tps[tps.i]]
+        }
```

```
+       } else {
+         mres.I = numeric()
+         mres.time = numeric()
+         mres.Imax = numeric()
+         for(Imax.i in 1:length(unique(Imax))){
+           mres = data.frame(lsoda(y = c(Ip = 0, Ic = 0),
+             times = sort(unique(c(seq(0, max(tps), steps), tps))),
+             func = mon.inf.lag.2p,
+             parms = c(rp = rp,
+               rc = rc,
+               Imax = unique(Imax)[Imax.i],
+               t0p = t0p,
+               t0c = t0c)))
+           mres.I = c(mres.I, mres$Ip+mres$Ic)
+           mres.time = c(mres.time, mres$time)
+           mres.Imax = c(mres.Imax,
+             rep(unique(Imax)[Imax.i],
+             length(mres$time)))
+         }
+         for(tps.i in 1:length(tps)){
+     Iout[tps.i]=mres.I[mres.time == tps[tps.i] & mres.Imax == Imax[tps.i]]
+         }
+       }
+     return(Iout)
+   }
```

ODE Functions (infection.models.r)

The monomolecular infection model (Raaijmakers et al., 2009; Paine et al., 2012) with an additional lag phase can be written as:

```
R> mon.inf.lag.1p = function(t, x, parms){
+     with(as.list(parms),{
+       if (t < t0) { dI = 0
+       } else { dI = r * (Imax - x[1]) }
+       return(list(dI))
+     })
+   }
```

The function contains three arguments, the first argument is the time `t`, the second argument, `x`, is a list of densities that occur in the differential equation system and the third argument, parms, is a list of constant parameters. We activate the headers of the parms list by using the `with()` function. Within the `with()` function the change of infections over time is calculated. We use an `if`/`else`-statement to discriminate between zero growth (before the first infections occur, `t < t0`) and positive new infections above this boundary. Lastly, the function returns a list containing the change of infected plants, `dI`. The two pathogen monomolecular infection model can be written as:

```
R> mon.inf.lag.2p = function(t, x, parms){
+     with(as.list(parms),{
+       if (t < t0p) {dIp = 0
+       } else { dIp = rp * (Imax - (x[1]+x[2])) }
+       if (t < t0c) {dIc = 0
+       } else { dIc = rc * (Imax - (x[1]+x[2])) }
+       return(list(c(dIp,dIc)))
+     })
+   }
```

The differences to `mon.inf.lag.1p()` are: Within the `with()` function, the change of infections over time is calculated following the two pathogen infection model (see main manuscript); the function returns a list containing the change in infected plants over time of the experimentally pathogen, `dIp`, and the naturally (control) occurring pathogen, `dIc`.

ACKNOWLEDGMENTS

We thank Katarina Fussmann, Myriam Hirt, Amrei Binzer, Andrew Barnes and Ulrich Brose for proof reading our manuscript and beta-testing the R-Code. All R-script files with corresponding data are added as online supporting information.

REFERENCES

Bolker, B. (2008). *Ecological Models and Data in R*. Princeton University Press, Princeton, N.J.

Bolker, B. and Team, R. D. C. (2016). ***bbmle**: Tools for general maximum likelihood estimation*. R package version 1.0.18.

Crawley, M. J. (2012). *The R Book*. John Wiley & Sons, Chichester, West Sussex, United Kingdom, 2nd edition.

Paine, C. E. T., Marthews, T. R., Vogt, D. R., Purves, D., Rees, M., Hector, A., and Turnbull, L. A. (2012). How to fit nonlinear plant growth models and calculate growth rates: an update for ecologists. *Methods in Ecology and Evolution*, 3(2):245–256.

R Core Team (2016). *R: A language and environment for statistical computing*. R Foundation for Statistical Computing, Vienna, Austria.

Raaijmakers, J. M., Paulitz, T. C., Steinberg, C., Alabouvette, C., and Moënne-Loccoz, Y. (2009). The rhizosphere: a playground and battlefield for soilborne pathogens and beneficial microorganisms. *Plant and Soil*, 321(1-2):341–361.

Soetaert, K. and Herman, P. M. (2009). *A Practical Guide to Ecological Modelling*. Springer Netherlands, Dordrecht, 1 edition.

Soetaert, K., Petzoldt, T., and Setzer, R. (2010). Solving differential equations in R: package **deSolve**. *Journal of Statistical Software*, 33(9):1–25.
